# Integration of a polygenic score into guideline-recommended prediction of cardiovascular disease

**DOI:** 10.1093/eurheartj/ehae048

**Published:** 2024-03-29

**Authors:** Ling Li, Shichao Pang, Fabian Starnecker, Bertram Mueller-Myhsok, Heribert Schunkert

**Affiliations:** Department of Cardiology, Deutsches Herzzentrum München, Technische Universität München, Lazarettstr. 36, Munich 80636, Germany; Deutsches Zentrum für Herz- und Kreislauferkrankungen (DZHK), Partner Site Munich Heart Alliance, Munich, Germany; School of Computation, Information and Technology, Technische Universität München, Munich, Germany; Department of Cardiology, Deutsches Herzzentrum München, Technische Universität München, Lazarettstr. 36, Munich 80636, Germany; Department of Cardiology, Deutsches Herzzentrum München, Technische Universität München, Lazarettstr. 36, Munich 80636, Germany; Deutsches Zentrum für Herz- und Kreislauferkrankungen (DZHK), Partner Site Munich Heart Alliance, Munich, Germany; Statistical Genetics, Max Planck Institute of Psychiatry, Munich, Germany; Institute of Translational Medicine, University of Liverpool, Liverpool, UK; Munich Cluster for Systems Neurology (SyNergy), Munich, Germany; Department of Cardiology, Deutsches Herzzentrum München, Technische Universität München, Lazarettstr. 36, Munich 80636, Germany; Deutsches Zentrum für Herz- und Kreislauferkrankungen (DZHK), Partner Site Munich Heart Alliance, Munich, Germany

**Keywords:** Cardiovascular disease, Polygenic risk score, SCORE2, Risk prediction, Primary prevention, Prevention guidelines

## Abstract

**Background and Aims:**

It is not clear how a polygenic risk score (PRS) can be best combined with guideline-recommended tools for cardiovascular disease (CVD) risk prediction, e.g. SCORE2.

**Methods:**

A PRS for coronary artery disease (CAD) was calculated in participants of UK Biobank (*n* = 432 981). Within each tenth of the PRS distribution, the odds ratios (ORs)—referred to as PRS-factor—for CVD (i.e. CAD or stroke) were compared between the entire population and subgroups representing the spectrum of clinical risk. Replication was performed in the combined Framingham/Atherosclerosis Risk in Communities (ARIC) populations (*n* = 10 757). The clinical suitability of a multiplicative model ‘SCORE2 × PRS-factor’ was tested by risk reclassification.

**Results:**

In subgroups with highly different clinical risks, CVD ORs were stable within each PRS tenth. SCORE2 and PRS showed no significant interactive effects on CVD risk, which qualified them as multiplicative factors: SCORE2 × PRS-factor = total risk. In UK Biobank, the multiplicative model moved 9.55% of the intermediate (*n* = 145 337) to high-risk group increasing the individuals in this category by 56.6%. Incident CVD occurred in 8.08% of individuals reclassified by the PRS-factor from intermediate to high risk, which was about two-fold of those remained at intermediate risk (4.08%). Likewise, the PRS-factor shifted 8.29% of individuals from moderate to high risk in Framingham/ARIC.

**Conclusions:**

This study demonstrates that absolute CVD risk, determined by a clinical risk score, and relative genetic risk, determined by a PRS, provide independent information. The two components may form a simple multiplicative model improving precision of guideline-recommended tools in predicting incident CVD.

## Structured Graphical Abstract

**Structured Graphical Abstract:**
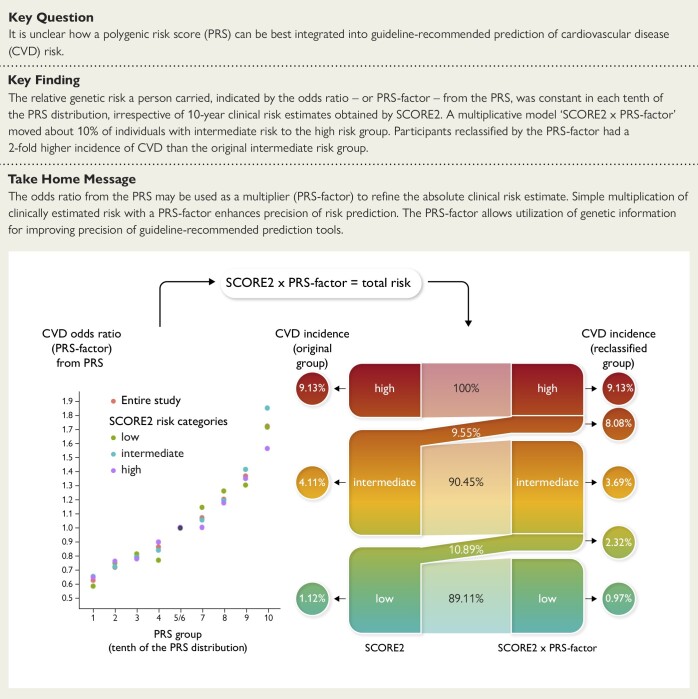
The left panel shows that relative risk related to polygenic disposition [1–10 tenth of the polygenic risk score (PRS) distribution] is independent of having a low, intermediate, or high clinical risk by SCORE2 in the UK Biobank. Thereby, the odds ratio from the PRS (relative risk) can be used as a genetic factor (PRS-factor) to multiply the absolute clinical risk estimate from SCORE2. If this is being done, ∼10% of the intermediate-risk group is being upgraded to a higher total risk (right panel). Subgroups that were genetically upgraded had incident event rates that were similar to those in the original high-risk groups and substantially increased the overall numbers of individuals correctly assessed to be at high risk. CVD, cardiovascular disease.

**See the editorial comment for this article ‘Polygenic risk scores for cardiovascular risk prediction: moving towards implementation into clinical practice?', by M. Christoffersen, https://doi.org/10.1093/eurheartj/ehae125.**

## Introduction

Genome-wide association studies (GWAS) have identified large numbers of genetic variants associated with cardiovascular disease (CVD) risk.^1^ Aggregated in the form of polygenic risk scores (PRS), these variants can be used for predicting the risk of coronary artery disease (CAD) or stroke.^1^ Indeed, individuals in the upper percentiles of such CAD-PRS were shown to have three times the risk for an event than the remainder of the population.^2^ Based on these findings, PRS are increasingly offered for individual risk assessment by commercial healthcare providers, although scientific societies do not recommend their use at present time.^3,4^ Some companies fuel expectations to determine risk solely on genetic grounds, whereas others offer concealed algorithms to determine the disease risk.^5^ In any case, wrongful interpretation of test results may potentially lead to unjustified management choices, unnecessary medical interventions, or unnecessary expenses.

European and American guidelines recommend risk assessment tools based on established risk factors to provide estimates for the probability of experiencing a CVD event within 5 or 10 years.^3,4,6^ A broad academic consensus allows translating these estimates into clinical recommendations including a healthier lifestyle or eventually medical therapy for those individuals at very high risk.^3,4,6,7^ While such guidelines for primary prevention of CVD are based on the estimation of *absolute clinical risk*, PRS report *relative genetic risk* in relation to the average of a given population, which thus far has limited their applicability. There is no recommended strategy to combine the two instruments.

In order to use a PRS as an adjunct to clinical risk assessment tools, i.e. SCORE2, the prediction tool recommended by the European Society of Cardiology (ESC),^8^ it is critical to clarify whether relative risks conferred by the genetic underpinnings are stable, irrespective of coexisting risk factors or the absolute risk a person carries from a clinical point of view. If so, once-in-a-lifetime assessment of the PRS would be sufficient to determine a factor (PRS-factor) measuring relative genetic risk, which subsequently would allow to multiply absolute risk obtained by SCORE2 or other clinical risk assessment tools. We, therefore, tested whether such PRS-factor confers a constant relative risk across the spectrum of clinical risk and analysed whether application of a PRS-factor adds to the precision of risk prediction in a clinically meaningful way for those at intermediate risk as estimated by clinical risk assessment tools. The study design is shown in Supplementary data online, *Figure S1*.

## Methods

### UK Biobank

UK Biobank (UKB) recruited ∼500 000 volunteers aged from 40 to 69 years at baseline through the United Kingdom National Health Service registers between 2006 and 2010.^9^ The UKB resource contains individual-level genotyping data and a rich variety of phenotyping and health-related data. The phenotype data were collected from several visits between 2006 and 2010, initial recruitment (2006–10), follow-up visit 1 (2012–13), follow-up visit 2 (2014+), or follow-up visit 3 (2019+).

We defined CAD and stroke cases based on codes of the International Classification of Diseases (ICD 9/10) or OPCS Classification of Interventions and Procedures version 4 (OPCS4).^10,11^ Participants who had neither ICD/OPCS codes nor self-reported codes of CAD/stroke were defined as controls. Lifestyle or other risk factors affecting CAD risk were extracted from UKB. Total cholesterol levels were calculated based on the Friedewald equation.^12^ We adjusted lipids and systolic blood pressure (SBP) levels in patients taking statin or blood pressure-lowering medicine.^13^ We defined a series of risk factors including older age (≥50 years), male sex, obesity [body mass index (BMI) ≥ 30 kg/m^2^], hypertension (SBP ≥ 140 mmHg), high cholesterol (total cholesterol > 6.18 mmol/L), and diabetes mellitus (types 1 and 2).

The genotyping data of UKB covers ∼96 million variants after imputation by referencing to the Haplotype Reference Consortium and UK10K haplotype resource. We restricted samples to a subset of European ancestry individuals. Series of quality control procedures were carried out on genotyping data using PLINK:^14^ (i) removing multi-allelic single nucleotide polymorphisms (SNPs), indels, and monomorphic; (ii) excluding variants that failed Hardy–Weinberg equilibrium test (HWE < 1e^−6^); (iii) removing variants of missing rate larger than 0.05 and imputation information smaller than 0.4; (iv) removing samples of missing rate large than 0.02 and kinship coefficient (P_HAT) larger than 0.125; and (v) excluding samples by sex discordance between genotyping data and reported sex.

### Framingham/Atherosclerosis Risk in Communities populations

The Framingham Heart (FH)^15^ and Atherosclerosis Risk in Communities (ARIC) studies^16^ were taken as a replication set for testing PRS-factor stability. The FH and Framingham Offspring studies began in 1971 with subsequent examinations every 3–8 years and included predominantly White individuals. The ARIC longitudinal study began in 1987 with the enrolment of adults aged 45 through 64 years. Three subsequent examinations were conducted approximately every 3 years until 1998.^16^

We applied the moderate-risk SCORE2 model to these studies from the USA. To be consistent with the ethnic composition of UKB, the participants of ARIC were limited to European Americans (80%). Only genetically independent samples were selected (PI_HAT < 0.125). Several decades of continuous follow-ups resulted in a very high CVD prevalence, so that participants were censored at the age of 60 years resulting in a reasonable population-level CVD prevalence (9.43%). We first did data pre-processing and PRS estimation for both studies separately. The sub-study GENEVA ARIC (phs000090.v7.p1) and sub-study SHARe FH (phs000342.v20.p13) containing genotype and phenotype data were downloaded from the dbGAP portal. The same quality control (QC) metrics used for the UKB were applied to the genotype data of Framingham/ARIC population. Finally, 8011 participants from ARIC and 2746 from FH provided necessary data (see Supplementary data online, *Table S1*).

### Clinical risk scores

Clinical risk was evaluated by SCORE2 which is widely used for predicting 10-year risk of first-onset CVD in Europe.^8^ The SCORE2 features a sex- and region-specific risk prediction model. Key inputs include sex, age, SBP, non-high-density lipoprotein (nonHDL) cholesterol, and current smoking status. SCORE2 classifies European countries into low-, moderate-, high-, and very-high-risk regions, the UK being a low-risk region.^8^ We mapped phenotype data from UKB to the reference table of a low-risk region in determining SCORE2-predicted risk.^8^ As suggested by SCORE2, participants were separated into three risk groups, low, intermediate, and high. We also studied QRISK3^6^ to test the consistency in clinical risk tools. To avoid the impacts of imbalanced sample sizes of clinical risk categories on PRS-factor, we also performed the same analyses for the tertile classification of SCORE2 and QRISK3.

### Polygenic risk score

The PRS, estimated by PRSice-2 (version 2.1.11),^17^ is a measure of accumulative weighted effects of risk alleles as shown in Formula (1). In this additive genetic model, *S_i_* is the effect size of the effective allele for SNP *i* and *G_ij_* coded as 0, 1, or 2 is the number of effective alleles for SNP *i* observed in participant *j*. Missing genotypes were replaced by the expected value, which was two times the risk allele frequency in the reference population.


(1) $$\mathrm{PR}S_{j}=\overset{n}{\underset{i}{\sum }}\;S_{i}\;\times G_{ij}.$$

Details of the selected variant list for calculating PRS were described elsewhere.^2^ Briefly, Khera *et al.*^2^ used GWAS summary data of CAD from CARDIoGRAMplusC4D (containing ∼185 000 participants) which did not include UKB^18^, to determine the effect sizes of risk alleles. Then, two different approaches were applied to build models of genome-wide PRS. One is the LDPred^19^ computational algorithm using different tuning parameters (the proportion of causal variants)—1.0, 0.3, 0.1, 0.03, 0.01, 0.003, and 0.001. The second approach was clumping and thresholding applied to a range of combinations of *P*-values (1.0, 0.5, 0.05, 5e^−4^, 5e^−6^, and 5e^−8^) and *r*^2^ (0.2, 0.4, 0.6, and 0.8). The one with the best discriminative accuracy was determined based on the maximal area under the receiver operating characteristic curve (AUC) in the logistic regression model of PRS and disease adjusted by age, sex, and the first four principal components of ancestry. Finally, the best model with 6.6 million SNPs was selected. We applied this list to compute CAD-PRS for the whole set of UKB. The logistic regression models were used to assess the relationship between PRS and CVD risk, with the adjustment for age, sex, and the first five PCs. By performing 10-fold cross-validations, the mean AUC was 0.732 (SD = 0.0067) (see Supplementary data online, *Figure S2*).

### Statistical analysis

The PRS deciles and conventional risk factors represent genetic (*G*) and clinical (*C*) factors to CVD risk, respectively. As described elsewhere,^20^ we next studied how genetic and environmental factors interactively affect CVD risk (*G* × *C*) using the logistic statistical model shown in Formula (2):


(2) $$Y=\mu +G\times C^{i}+\varepsilon ,$$

where *Y* is the binary outcome of CVD, *μ* represents a constant, *ε* serves as residual error that cannot be controlled, and *i* represents *i*-way interaction effects of clinical exposures on the underlying scale. The best models were selected by Akaike information criterion (AIC).^21^ All association models were adjusted for the first five principal components. Formula (2) was also applied to test for interactions between clinical risk scores of CVD and the CAD-PRS.

To estimate the PRS-factor, we separated participants equally into deciles based on the PRS distribution. The fifth and sixth tenths were merged as a reference group. Then, we computed relative genetic risks, namely odds ratios (ORs), for each PRS tenth by comparing them with the reference group. Besides ORs in the entire cohort, we also computed the ORs of CVD within each CAD-PRS tenth for participants carrying various conventional risk factors. As shown in Formula (3), we divided participants in each PRS group *i* exposed to risk factor *j* into 10 equal subgroups to calculate CVD ORs. In this formula, *D_ij_* is the number of CVD cases in subgroup *ij* and *H_ij_* is the number of controls in that subgroup. Within each PRS group, we performed analysis of variance (ANOVA) test to check differences in mean ORs between the entire cohort and subgroups by risk factors:


(3) $$\;\;\;\;\;\;\;\;\;\;\;\;\;\mathrm{Odds}\;\mathrm{rati}o_{ij}=\;\frac{D_{ij}/D_{5/6,j}}{H_{ij}/H_{5/6,j}}.$$

Similar operations were applied to clinical risk categories determined by SCORE2/QRISK3. Within each PRS tenth, the clinical risk categories were further divided into five equal subgroups. Then, we calculated ORs for each subgroup based on Formula (3) and tested the difference in mean ORs between the entire cohort and clinical risk categories using ANOVA test. The *P*-values were adjusted by the false discovery rate (FDR) approach.

For the Framingham/ARIC populations, the PRSs were selected by the highest AUC from multiple LDpre2d models or ‘C + T’ models (see Supplementary data online, *Table S2*). Within each study, participants were equally split into 10 PRS groups (tenths). Then, we combined the PRS classification from both studies together to test the stability of PRS-factor. The fifth/sixth PRS tenths were taken as the reference group for calculating ORs. Within each PRS tenth, samples were equally separated into four groups. Then ANOVA test was applied to test the significance of the difference in mean CVD OR between the entire cohort and subgroups of traditional risk factors or clinical risk categories.

At last, we also constructed a new model for estima6ting an individual’s total risk by multiplying the absolute clinical risk estimated by SCORE2 or QRISK3 with relative genetic risk measured as PRS-factor, shown in Formula (4):


(4) SCORE2×(PRS-factor)=totalrisk.

We used Cox proportional hazards model for analysis of incident events on subset of UKB and ARIC/Framingham participants developing myocardial infarction or stroke after initial assessment. Values of the C-index or concordance index^22^ were compared between two risk prediction models: (i) CVD ∼ SCORE2 and (2) CVD ∼ SCORE2 × (PRS-factor). Net reclassification index (NRI) was also used to compare performance of the two models. Both C-index and NRI were estimated as mean of 1000 bootstrapped samples.

## Results

### Study population

The study design is shown in Supplementary data online, *Figure S1*. Among 432 981 participants of UKB with genotype and phenotype data, we identified 27 532 CVD cases (CAD 19 617, stroke 6757, or both 1158) and 405 449 controls. The main characteristics are shown in *Table 1*. The CVD prevalence was significantly higher in subgroups exposed to traditional risk factors than the entire cohort and higher in men than in women, which was the same in the Framingham/ARIC populations (see Supplementary data online, *Table S1*). It markedly increased across the clinical risk categories determined by SCORE2, being 7.54-fold times higher in the high-risk as compared with the low-risk category (*Table 1*).

**Table 1:** Cardiovascular disease prevalence by traditional risk factors and risk categories of SCORE2

Group	Women *n* (CVD%)	Men *n* (CVD%)	Total (CVD %)
Entire	237 161 (3.72)	195 820 (9.55)	432 981 (6.36)
Age ≥ 50	181 607 (4.52)	149 447 (11.41)	331 054 (7.63)
Diabetes mellitus	13 580 (12.96)	18 569 (22.46)	32 149 (18.45)
Current smoking	20 699 (6.33)	24 094 (11.61)	44 793 (9.17)
Obesity (BMI ≥ 30 kg/m^2^)	55 109 (5.25)	48 326 (12.19)	103 435 (8.49)
Hypertension (SBP ≥ 140 mmHg)	94 315 (5.6)	102 499 (12.05)	196 814 (8.96)
High cholesterol (> 6.18 mmol/L)	89 672 (4.7)	66 378 (10.63)	156 050 (7.22)
SCORE2 low	103 148 (1.89)	25 504 (3.1)	128 652 (2.13)
SCORE2 intermediate	61 043 (6.15)	89 836 (8.63)	150 879 (7.63)
SCORE2 high	1347 (12.92)	25 234 (16.24)	26 581 (16.07)

CVD, cardiovascular disease prevalence is shown (in per cent); BMI, body mass index; SBP, systolic blood pressure.

### PRS-factor Stability relative to traditional risk factors

We grouped participants into tenths of the PRS, which follows a normal distribution, and observed that CVD prevalence in the highest tenth was 2.57-fold higher than in the first tenth (see Supplementary data online, *Figure S2*). Within a PRS tenth, subgroups exposed to modifiable and non-modifiable risk factors have higher CVD prevalence (see Supplementary data online, *Figure S3*).

**Figure 1:**
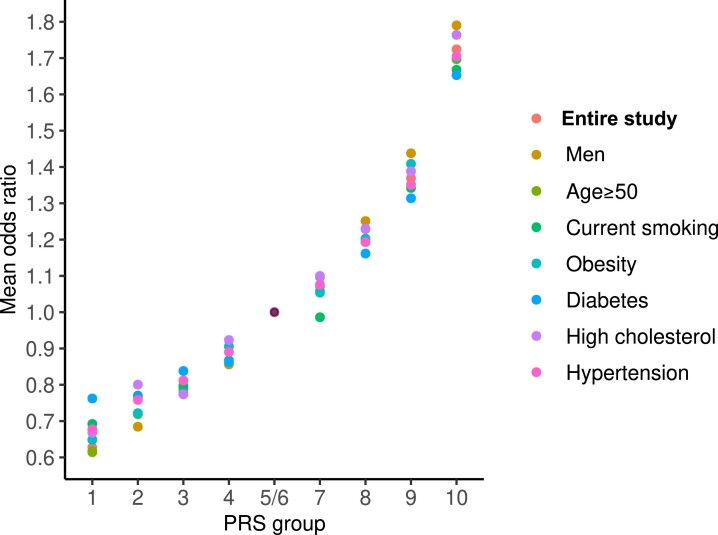
Odds ratios (PRS-factors) in the entire study population and subgroups carrying traditional risk factors. The figure shows the distribution of the PRS-factor, measured as mean CVD ORs, along PRS tenths in UK Biobank. The fifth/sixth groups were taken as the reference (OR = 1.0). The figure shows within one tenth of the PRS little (non-significant) variation between the entire set and subgroups carrying traditional risk factors. OR, odds ratio; CVD, cardiovascular disease; PRS, polygenic risk score

We then built a genetic reference group by merging the fifth and sixth tenths (OR = 1), which had a CVD prevalence of 6.12% close to the average of the entire group (6.36%). Next, we calculated CVD ORs (PRS-factor) for the other eight tenths. Within each of these PRS tenths, we compared the ORs obtained in the entire cohort and subgroups exposed to various clinical risk factors. As shown in *Figure 1*, with an increasing PRS, the ORs for CVD increased exponentially in the entire cohort (*R^2^* = 0.98, *P* = 1.4e^−7^), consistent with our previous findings.^20^ Here, we show by ANOVA test that within each of the PRS tenths, there was no significant difference in mean ORs (FDR > 0.05) among the entire cohort and subgroups exposed to various modifiable risk factors (*Figure 1* and Supplementary data online, *Table S3*). Similar results were observed in the Framingham/ARIC populations (*n* = 10 757) (see Supplementary data online, *Table S3* and *Figure S4*).

### PRS-factor Stability relative to SCORE2 levels

**Figure 2:**
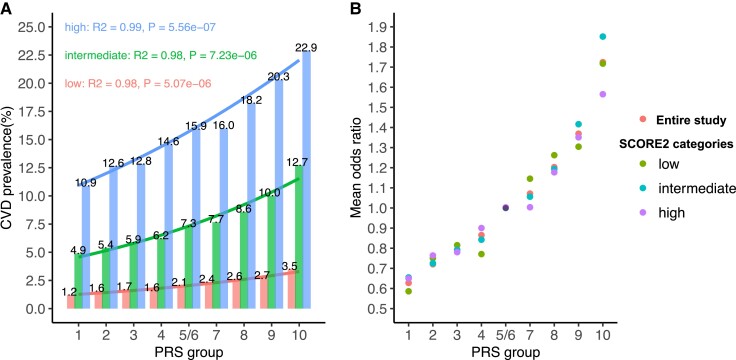
Cardiovascular disease prevalence and PRS-factors in the entire study and risk categories of SCORE2. (*A*) Cardiovascular disease prevalence along PRS tenths in clinical risk categories of SCORE2 (low, intermediate, and high). The distribution of CVD prevalence in SCORE2 risk categories fits into a Logit model with *R*^2^ > 0.9. (*B*) Cardiovascular disease ORs in risk categories of SCORE2 by PRS tenths; the fifth/sixth groups were taken as reference (OR = 1.0). The figure shows within one tenth of the PRS little (non-significant) variation of the ORs, irrespectively of SCORE2 risk categories, albeit the CVD prevalence differs significantly between low- and high-risk categories (*A*). OR, odds ratio; CVD, cardiovascular disease; PRS, polygenic risk score

Within each SCORE2 risk category, the association between CVD prevalence and PRS fitted into Logit models (*Figure 2*), in line with previous observations on the entire cohort.^21^ The steepness of regression curves between CVD prevalence and PRS tenths increased from the low- to high-risk SCORE2 categories (*Figure 2A*). However, the CVD ORs (PRS-factor) related to each PRS tenth as compared with the respective reference group (Groups 5/6) were consistent in the three clinical risk categories of SCORE2 (*Figure 2B* and *Table 2*), which was replicated in the Framingham/ARIC populations (see Supplementary data online, *Table S4* and *Figure S5*). Similar findings were observed in UKB in clinical risk categories of QRISK3 (see Supplementary data online, *Table S5* and *Figure S6*) which demonstrate the stability of ORs (PRS-factor) across risk categories in clinical risk estimation tools. To avoid an impact of unequal sample size, we also grouped individuals into tertiles of SCORE2 and QRISK3, showing that the ORs for CVD within any PRS groups were consistent irrespective of the highly variable clinical level of risk (see Supplementary data online, *Table S6* and *Figure S7*).

**Table 2:** Odds ratios (PRS-factors) within polygenic risk score tenths are similar across SCORE2 risk categories

PRS groups	Mean CVD ORs in the entire cohort	Mean CVD ORs by SCORE2 risk categories (95% CI)			ANOVA test				
	(95% CI)	Low	Intermediate	High	Sum Sq	Mean Sq	*F* value	Pr(>F)	FDR
1	0.63 (0.6–0.65)	0.59 (0.42–0.75)	0.65 (0.61–0.7)	0.65 (0.57–0.73)	0.02	0.01	0.41	0.75	1.00
2	0.72 (0.7–0.74)	0.75 (0.63–0.87)	0.72 (0.69–0.76)	0.76 (0.68–0.85)	0.01	0.00	0.29	0.83	1.00
3	0.79 (0.76–0.82)	0.82 (0.71–0.92)	0.79 (0.76–0.82)	0.78 (0.67–0.9)	0.00	0.00	0.12	0.95	1.00
4	0.87 (0.83–0.9)	0.77 (0.68–0.86)	0.84 (0.8–0.88)	0.9 (0.8–1)	0.05	0.02	2.23	0.13	0.37
5/6	1	1	1	1					
7	1.07 (1.03–1.11)	1.15 (1.03–1.26)	1.06 (1.03–1.08)	1 (0.94–1.07)	0.05	0.02	2.75	0.08	0.35
8	1.2 (1.18–1.23)	1.26 (1.18–1.34)	1.19 (1.16–1.23)	1.18 (1.12–1.24)	0.02	0.01	1.68	0.21	0.48
9	1.37 (1.33–1.41)	1.3 (1.17–1.43)	1.42 (1.36–1.47)	1.35 (1.22–1.48)	0.03	0.01	0.87	0.48	0.86
10	1.72 (1.67–1.78)	1.72 (1.59–1.84)	1.85 (1.79–1.91)	1.57 (1.41–1.72)	0.21	0.07	4.49	0.02	0.16

The first column shows the PRS tenths of which the fifth/sixth groups were taken as the reference (OR = 1). The middle part shows CVD ORs in the entire cohort and three risk categories of SCORE2 within respective PRS tenths. The right part is the result of the ANOVA test (degree of freedom = 3) for the difference of OR between the entire cohort and SCORE2 risk categories—showing no statistical interaction. PRS, polygenic risk score; OR, odds ratio; CVD, cardiovascular disease; CI, confidence interval; Sum Sq, the sum of squares of total variation between the group means and the overall mean; Mean Sq, the mean of the sum of squares; *F* value, the test statistic from the *F* test; Pr(>F), the *P*-value of the *F* statistic; FDR, false discovery rate.

### Independent effects between polygenic risk score and SCORE2 on cardiovascular disease risk

We further analysed statistically the independence of genetic (G) and clinial (C) risk on CVD risk by studying their interactive effects. The two-way interactions (*G* × *C*^2^) with the lowest AIC revealed that all modifiable clinical risk factors and PRS had independent effects on CVD risk. Regarding the non-modifiable risk factors, i.e. age and sex, we revealed from the two-way interaction model statistically significant (*P* < 0.05) interactions for ‘PRS:Men’ (*Table 3*). Importantly, studying clinical risk categories of SCORE2, we observed no such one-way interaction (*G* × *C*) with the PRS (*Table 3*). The same results were observed for clinical risk categories determined by QRISK3, as well as the tertile groups classified by both tools (see Supplementary data online, *Table S7*). These findings suggest that the relative polygenic contribution is largely independent of absolute clinical risk as assessed by a recommended clinical score. Thus, the ORs derived from a PRS model can be converted into a PRS-factor which—multiplied with the absolute clinical risk from the score—may allow a refined estimate of total CVD risk.

**Table 3:** Interactive effects on cardiovascular disease risk between polygenic risk score and clinical risk factors/SCORE2

Interaction	Beta	Std. error	*z* value	Pr(>|z|)
PRS with modifiable risk factors				
High cholesterol (> 6.18 mmol/L)	0.052	0.025	2.126	0.033
Hypertension (SBP ≥ 140 mmHg)	0.023	0.026	0.897	0.37
Obesity (BMI ≥ 30 kg/m^2^)	0.005	0.028	0.174	0.862
Diabetes mellitus	−0.041	0.035	−1.152	0.249
Smoking	−0.017	0.03	−0.577	0.564
PRS with non-modifiable risk factors				
Men	0.111	0.025	4.422	9.78E-06
Age ≥ 50	0.037	0.025	1.468	0.142
PRS with clinical risk categories of SCORE2				
SCORE2	−0.008	0.005	−1.523	0.128

The first column shows interactive items [clinical risk factors/SCORE2 (*C*)]. The ‘Beta’ column shows the interactive effect size with PRS tenths [genetic risk factor (*G*)] by applying a logistic regression model. ‘Pr(>|z|)’ is the statistical test of the interactive effect, which was non-significant, with the exception of male gender. CVD, cardiovascular disease; PRS, polygenic risk score; SBP, systolic blood pressure; BMI, body mass index.

### Risk reclassification by ‘SCORE2 × PRS-factor’

We next constructed a risk prediction model by multiplying an individual’s absolute clinical risk according to SCORE2 with the respective PRS-factor, ‘SCORE2 × PRS-factor’. This model was applied to 297 201 participants free of CVD at the initial assessment. Compared with SCORE2 alone, ‘SCORE2 × PRS-factor’ modestly increased C-index by 0.9% (*P* = 1.6e^−12^) and net classification index (NRI) by 0.063 (95% CI 0.059–0.067) as shown in Supplementary data online, *Table S8*, findings that were close to a study by Lu *et al.*^23^

**Figure 3:**
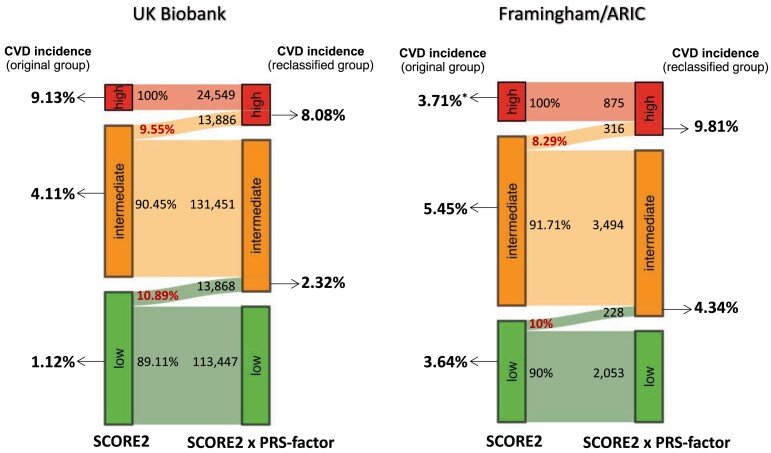
Sankey diagrams visualizing reclassification by PRS-factor. The figure shows the subgroups which were genetically upgraded (PRS-factor > 1) from a lower risk category of SCORE2 to a higher risk category based on ‘SCORE2 × PRS-factor’ in UK Biobank (left) and Framingham/ARIC (right) populations. The left side of two Sankey plots shows CVD incidence of the original groups of SCORE2, and the right side shows CVD incidence of subgroups genetically upgraded by ‘SCORE2 × PRS-factor’. CVD, cardiovascular disease. *The low incidence rate of high-risk category of SCORE2 in Framingham/ARIC populations is because of a high number of prevalent cases (*n* = 130) that had to be excluded for the analysis of incident events and a small sample size (*n* = 875)

Compared with the risk groups classified by SCORE2 alone, the multiplicative model reclassified a sizeable proportion of participants (*Table 4*, *Figure 3*, and Supplementary data online, *Table S9*). The subgroups moved by the PRS-factor to a higher risk category had a higher CVD incidence than the subgroup kept in the original risk category. Vice versa, the subgroups with lowered risk by the PRS-factor had a lower CVD incidence than the original class. In detail, the PRS-factor reclassified 33.77% of high-risk participants into intermediate risk, who indeed had a lower CVD incidence (6.87%) than the original high risk category (9.13%). Since the PRS-factor comes with a 2.73-fold gradient of polygenic risk, we did not observe that a person was moved from low to high, or *vice versa*, from high to low total risk (*Table 4*).

**Table 4:** Risk reclassification by ‘SCORE2 × PRS-factor’

SCORE2 vs. ‘SCORE2 × PRS-factor’	*n*	CVD incidence (%)^a^	Prevalence of risk factors (%)^b^						
			Diabetes	Smoking	Obesity	Hypertension	High cholesterol	Male	Age ≥ 50
Original low (100%)^c^	127 315^c^	1.12^c^	3.65^c^	3.09^c^	20.70^c^	19.81^c^	33.22^c^	19.73^c^	66.67^c^
Low (89.11%)^d^	113 447^d^	0.97^d^	3.48^d^	2.82^d^	20.34^d^	18.39^d^	32.06^d^	18.59^d^	66.34^d^
Intermediate (10.89%)^e^	13 868^e^	2.32^e^	5.05^e^	5.28^e^	23.64^e^	31.42^e^	42.70^e^	29.03^e^	69.33^e^
Original intermediate (100%)^c^	145 337^c^	4.11^c^	8.14^c^	12.28^c^	25.94^c^	62.57^c^	48.21^c^	59.20^c^	83.48^c^
Low (19.58%)^d^	28 453^d^	2.29^d^	6.01^d^	7.88^d^	23.49^d^	51.6^d^	43.44^d^	50.35^d^	87.06^d^
Intermediate (70.87%)^e^	102 998^e^	4.08^e^	8.33^e^	13.07^e^	26.43^e^	64.12^e^	48.80^e^	60.99^e^	81.06^e^
High (9.55%)^f^	13 886^f^	8.08^f^	11.08^f^	15.47^f^	27.34^f^	73.58^f^	53.62^f^	64.06^f^	94.04^f^
Original high (100%)^c^	24 549^c^	9.13^c^	13.66^c^	34.64^c^	26.80^c^	91.63^c^	52.49^c^	94.80^c^	97.36^c^
Intermediate (33.77%)^e^	8291^e^	6.87^e^	11.95^e^	28.39^e^	25.77^e^	90.15^e^	49.91^e^	95.19^e^	98.05^e^
High (66.23%)^f^	16 258^f^	10.28^f^	14.53^f^	37.83^f^	27.32^f^	92.39^f^	53.81^f^	94.60^f^	97.01^f^

The data show that individuals reclassified by the PRS-factor to a higher or lower risk category had incidence rates close to those originally classified in respective risk categories.

PRS, polygenic risk score; CVD, cardiovascular disease; PRS, polygenic risk score; CVD, cardiovascular disease.

^a^Incidence rate of events in those initially free of CVD.

^b^Prevalence of risk factors at initial assessment.

^c^‘Original’ classification by SCORE2 only.

^d^Data obtained after multiplying SCORE2 with PRS-factor (low).

^e^Data obtained after multiplying SCORE2 with PRS-factor (intermediate).

^f^Data obtained after multiplying SCORE2 with PRS-factor (high).

From a clinical perspective, we focused on the intermediate-risk category (*n* = 145 337), of whom 13 886 or 9.55% were moved by the PRS-factor to high risk (*Table 4* and *Figure 3*). Thereby, the number of individuals considered to be at high risk increased by 56.6% as compared with the classification by SCORE2 alone. The incidence of CVD events in individuals moved from intermediate to high risk by the PRS-factor was 8.08%, which was close to the incidence in the original high-risk group based on SCORE2 alone (9.13%) and 1.98-fold of those who remained in the intermediate-risk group (4.08%; *n* = 102 998). Similar observations were made for the QRISK3 model (see Supplementary data online, *Table S9*) and in the combined ARIC/Framingham populations (*Figure 3*). Thus, the PRS-factor appears to have the most clinical relevance for individuals at intermediate clinical risk—but at high genetic risk (PRS-factor > 1)—who may be considered for an intensified preventive treatment.

## Discussion

Common genetic variants with small effects on CVD risk can be aggregated in the form of polygenic scores which have been shown to associate with the prevalence of CAD or stroke.^1,2^ It is undecided how this genetic information is best integrated into guideline-recommended risk prediction tools. Here, we show that the *relative* genetic risk along the distribution curve of a PRS is fairly stable regardless of prevalent traditional risk factors or the overall *absolute* clinical risk a person carries. Thereby, the relative genetic and absolute clinical risks a person carries work independently on affecting individual total CVD risk. The stability and independence of polygenic risk allow to generate a PRS-factor that can be easily integrated into established risk assessment tools and add currently unexplored information from a given person which may inform to guideline recommendations.

Indeed, application of such multiplicative model improved risk prediction in a quantitatively and clinically relevant way. Specifically, the simple multiplicative model ‘SCORE2 × PRS-factor’ applied to participants at intermediate risk by SCORE2, who represent about half of UKB, increased the number of individuals to be considered at high risk by 56.6%. Individuals who were moved by the PRS-factor from intermediate to high risk experienced CVD events about twice as often as those who were grouped to intermediate risk based on SCORE2 alone (*Structured Graphical Abstract*). Thus, the inclusion of relative genetic risk as determined by the PRS—multiplying the clinical risk for a CVD event as predicted by SCORE2—added clinically relevant precision in selected individuals from the UKB.

The use of PRS is not recommended by current guidelines.^3^ Indeed, given the high frequency and random distribution of risk alleles, their number displays relatively little variability within a population, which is consistent with the genetic sampling theory.^24^ Thereby, PRS offers only small improvements of the AUCs for incident CVD events, when studied in the entire population.^21,24–26^ In extension of this notion, we show that the majority of the population has a PRS-factor close to 1.0 resulting in only small changes in the expected future event rates. However, the multiplicative effects of risk alleles result in an exponential increase of relative risk such that subjects in the highest tenth have PRS-factors close to 2.^20^

Importantly, knowledge of a PRS results is only meaningful in the context of other risk factors. Specifically, a high PRS may increase the absolute 10-year risk by as little as 0.6%, e.g. in a healthy woman, or by more than 10% in a middle-aged smoker (see Supplementary data online, *Figure S3*). Thus, the clinical relevance of being in a high percentile of the PRS depends largely on the outcome from the clinical risk assessment, such as obtained by SCORE2, and should not be seen in isolation.^21^ Indeed, our data suggest that using a PRS without recognition of the overall CVD risk may be even misleading (*Figure 2A*). This should be kept in mind when PRS are marketed as sole source for predictive information by some ‘over-the-counter’ products. On the other hand, there does not seem to be the need for complex (black box) algorithms, which are likewise commercially available and translate polygenic risk into a clinical recommendation. Instead, the use of PRS-factor as an adjunct to a guideline-recommended prediction tool like SCORE2 may help to keep the focus on the most imminent issues in primary prevention, e.g. modifiable risk factors, since their effects are amplified by polygenic risk and their treatment is already specified in the guidelines.^25^

Our study comes with a number of limitations. To avoid bias, we built the CAD-PRS excluding data from UKB, which precluded the use of GWAS data for stroke and might have reduced the precision for studying CVD risk (CAD plus stroke). However, our principal aim was to elucidate whether the ORs obtained from a PRS are comparable at different levels of overall risk, which should not be affected by this strategy. Moreover, there is an overlap of genetic risk loci between CAD and stroke^26^ and the majority of CVD events in this population was related to CAD, such that the use of the CAD-PRS (rather than a combined CAD/stroke PRS) may have had little effect on our overall results. Nevertheless, larger and ethnically more diverse GWAS covering stroke may further refine the polygenic risk estimates.^27^ Second, we wanted to avoid that risk estimates on clinical grounds are being ‘downgraded’ by a low PRS. Therefore, we would advise to apply the PRS-factor for clinical counselling only when it is larger than 1 (*Structured Graphical Abstract*). Obviously, this strategy needs further evaluation. Third, it needs to be determined whether the generalizability of a PRS result across the spectrum of clinical risk can be extrapolated to individuals outside the age range or the ethnic group studied in UKB and Framingham/ARIC. Finally, although we show that PRS and SCORE2 act independently on clinical risk of CVD, the generalizability to other common diseases and additional environmental risk factors needs to be verified.

## Conclusions

Across the entire spectrum of clinical risk, as calculated by the ESC guideline-recommended prediction tool SCORE2, we observed—for a given position in the distribution curve of a CAD-PRS—little variability by which polygenic risk multiplied absolute CVD risk. Thus, the polygenic risk an individual carries may be used in the form of PRS-factor to enhance the precision of risk estimates obtained by conventional measures. Applying the PRS-factor substantially increased the number of subjects correctly considered to be at high risk, making the strategy attractive from a clinical point of view as a refinement of recommended instruments of CVD risk assessment.

## Supplementary Material

ehae048_Supplementary_Data

## Declarations

### Data Availability

Genome-wide association studies summary data used for building PRS are available at https://s3.amazonaws.com/broad-portal-resources/CVDKP/Lifetime_Risk_SNPs.txt. The use of UK Biobank data was authorized under application ID 25214. The use of Framingham (phs000342.v20.p13) and ARIC (phs000090.v7.p1) data was authorized by dbGAP under application ID 6271. QRISK3 package is available at https://cran.r-project.org/web/packages/QRISK3/index.html.

### Pre-registered Clinical Trial Number

There is nothing to declare about the clinical trial number because it’s not a clinical trial study.
