# Supplementary material for: Integration of a polygenic score into guideline-recommended prediction of cardiovascular disease
Source: Eur Heart J. 2024 Mar 29;45(20):1843–52. doi: 10.1093/eurheartj/ehae048 (PMC11129792; doi:10.1093/eurheartj/ehae048)
Supplement: ehae048_Supplementary_Data [file ehae048_supplementary_data.zip › Supplementary files_1128.docx]

# Supplementary Table list

Table S1. CVD prevalence by traditional risk factors and risk categories of SCORE2 (Framingham/ARIC).

Table S2. Polygenic risk score training for Framingham/ARIC population: 'Clumping + Thresholding' and LDpred2.

Table S3. ANOVA test for difference in mean ORs between the entire study and subgroups of risk factors (UK Biobank and Framingham/ARIC).

Table S4. ANOVA test for difference in mean ORs between the entire study and risk categories of SCORE2 (UK Biobank and Framingham/ARIC).

Table S5. ANOVA test for difference in mean ORs between the entire study and clinical risk categories (low, intermediate, and high) of QRISK3 (UK Biobank).

Table S6. ANOVA test for difference in mean ORs between the entire study and clinical risk tertile groups of SCORE2 and QRISK3 (UKB).

Table S7. Interactive effects between PRS and SCORE2/QRISK3 on CVD risk.

Table S8. Model performance, C-index and NRI.

Table S9. Risk reclassification by SCORE2/QRISK3 x PRS-factor models.

# Supplementary Figure list

Figure S1. Study design.

Figure S2. PRS distribution of UK Biobank.

Figure S3. CVD prevalence by PRS tenths in subgroups of risk factors.

Figure S4. Distribution of PRS-factor in the entire study and subgroups carrying traditional risk factors in the Framingham/ARIC populations (n = 10,757).

Figure S5. Distribution of PRS-factor in the entire study and risk categories of SCORE2 in the Framingham/ARIC populations (n = 10,757).

Figure S6. CVD prevalence and PRS-factor in entire study and risk categories of QRISK3.

Figure S7. CVD prevalence and PRS-factor in clinical risk tertiles of SCORE2 and QRISK3.

## Figure S1.

**Figure S1. Study design.** We first constructed CAD PRS based on GWAS summary data from CARDIoGRAMplusC4D for 432,981 participants of the UK Biobank. Traditional risk factors for CVD were also extracted from UK Biobank. We estimated the clinical risk using the low-risk model of SCORE2. Within each PRS tenth, we next tested the stability of PRS-factor measured as CVD ORs by PRS groups between the entire study and subgroups defined by clinical risk factors or scores. The observation suggests a simple multiplicative model in which the ORs can be applied as a multiplier to clinical risk estimates (SCORE2 × PRS-factor = total risk) to refine current clinic risk estimates with polygenic factors. Same pipeline was also applied to QRISK3 as an alternative risk score. The replication analysis was performed in the combined Framingham/ARIC populations (*n* = 10,757). PRS, polygenic risk score; OR, odds ratio; CAD, coronary artery disease; CVD, cardiovascular disease (CVD, i.e. CAD or stroke); ARIC, the Atherosclerosis Risk in Communities.

## Figure S2


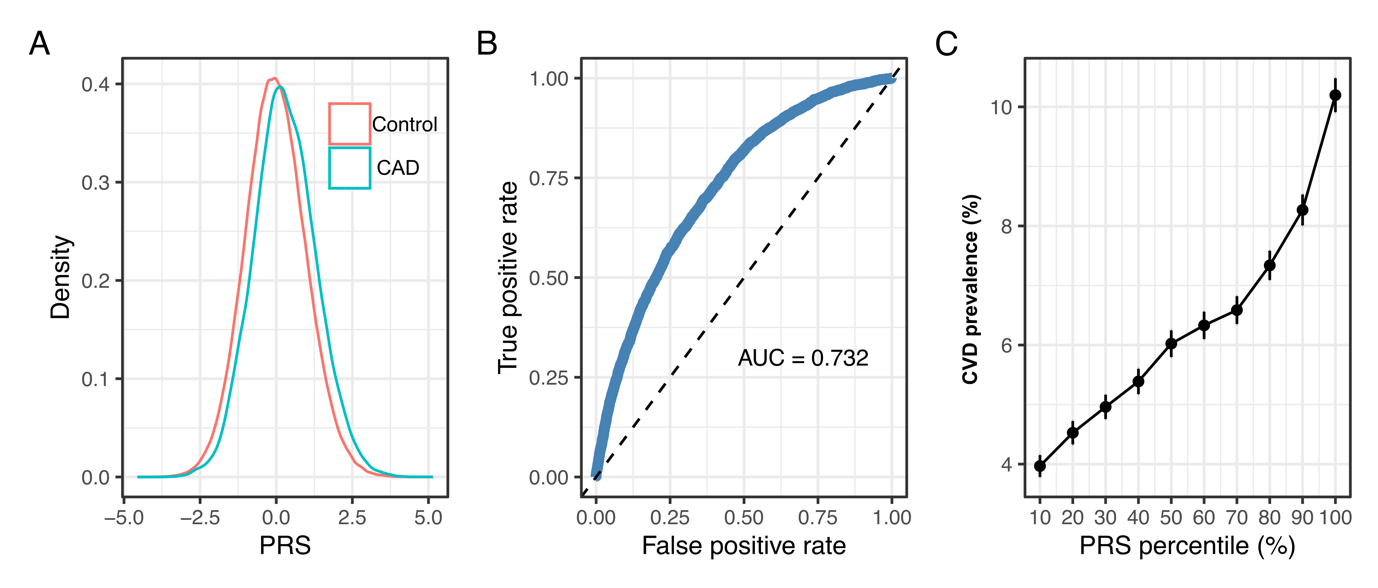


**Figure S2. PRS distribution of UK Biobank.** A) Normal distribution of CAD PRS for participants of UK Biobank. CAD and controls are marked in blue and red respectively. B) Receiver operating characteristic curve of the PRS for the prediction of CVD The was estimated as the average of 10-fold cross-validation. C) Distribution of CVD prevalence along with the PRS percentile. PRS, polygenic risk score; CAD, coronary artery disease; CVD, cardiovascular disease; AUC, area under the Receiver Operating Characteristic Curve.

## Figure S3

**Figure S3. CVD prevalence by PRS tenths in subgroups of risk factors.** Risk factors (RFs) are classified as nonmodifiable factors, like sex and age, and modifiable factors like diabetes, high cholesterol, etc. The CVD prevalence increases with the increase of PRS. Within a PRS tenth, the subgroups with risk factors have higher prevalence than subgroups without. The male groups have higher CVD prevalence than the respective female subgroups. CVD, cardiovascular disease; PRS, polygenic risk score.

## Figure S4

**Figure S4. Distribution of PRS-factors in the entire study and subgroups carrying traditional risk factors in the Framingham/ARIC populations (n = 10,757).** The figure shows the distribution of the PRS-factor, measured as mean CVD ORs, within PRS tenths in the replication set. The fifth/sixth groups were taken as the reference (OR = 1.0). The figure shows little (non-significant) variation of PRS-factor between the entire set and subgroups carrying traditional risk factors. OR, odds ratio; CVD, cardiovascular disease; PRS, polygenic risk score.

## Figure S5


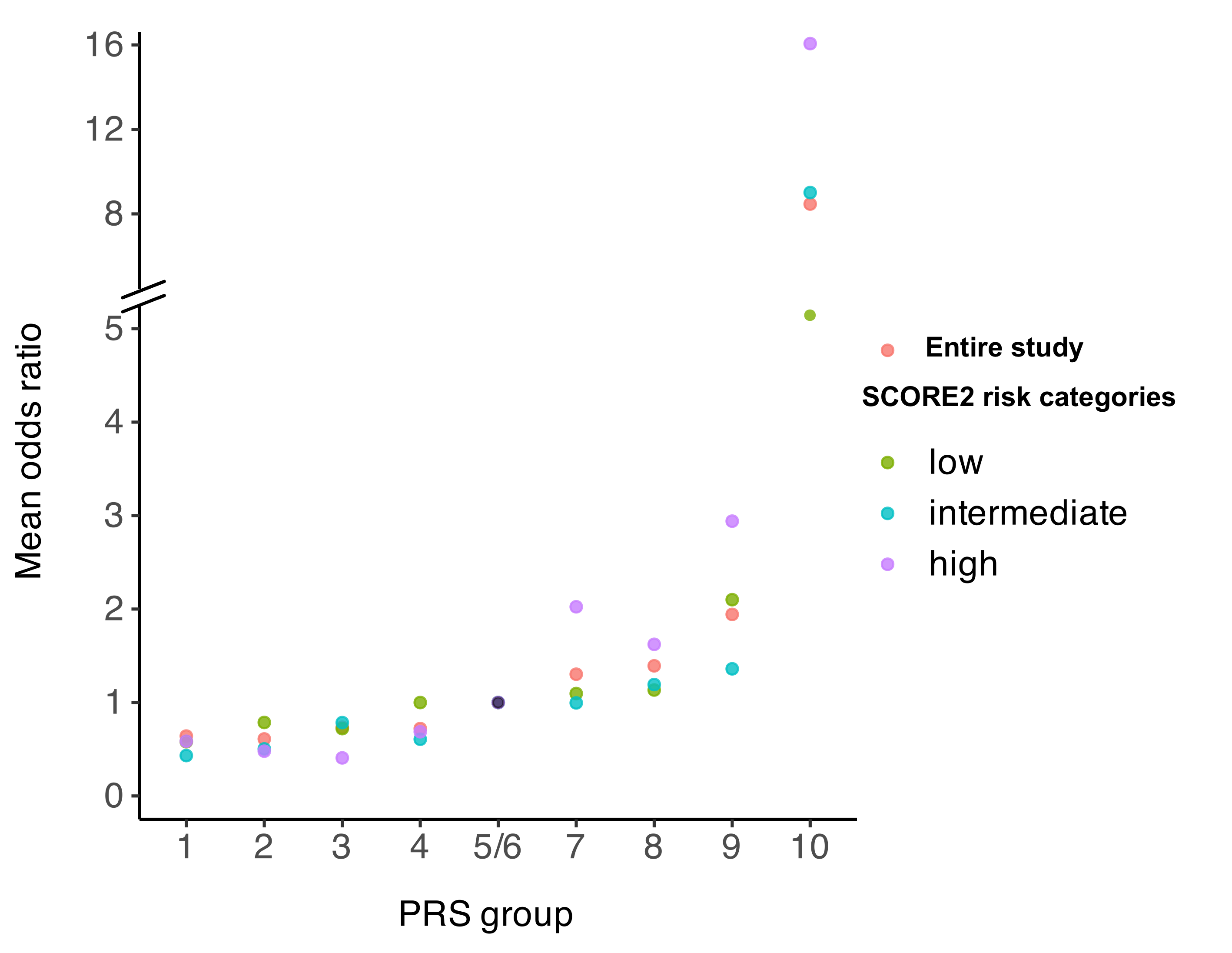


**Figure S5. Distribution of PRS-factors in the entire study and risk categories of SCORE2 in the Framingham/ARIC populations (n = 10,757).** The figure shows the distribution of the PRS-factor, measured as mean CVD ORs along the PRS tenths in the replication set. The fifth/sixth groups were taken as the reference (OR = 1.0). The figure shows little (non-significant) variation of PRS-factor between the entire set and clinical risk categories of SCORE2. OR, odds ratio; CVD, cardiovascular disease; PRS, polygenic risk score.

## Figure S6


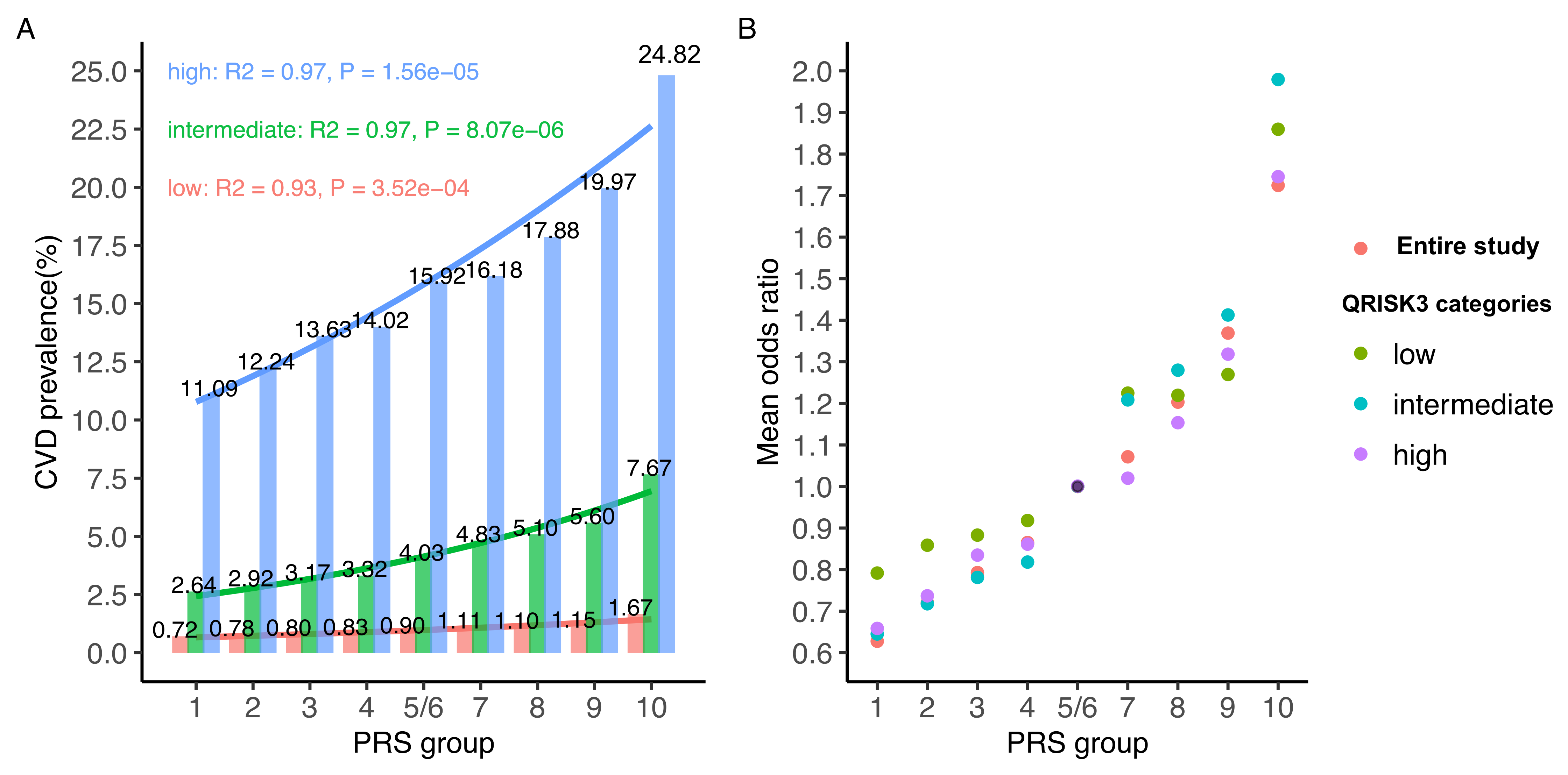


**Figure S6**. **CVD prevalence and PRS-factors in entire study and risk categories of QRISK3.** A) CVD prevalence along PRS tenths in clinical risk categories of QRISK3 (low, intermediate, and high). The distribution of CVD prevalence in QRISK3 risk categories fits into a Logit model with R^2^ > 0.9. B) CVD ORs in risk categories of QRISK3 by PRS tenths of which fifth/sixth groups were taken as reference (OR = 1.0). The figure shows little (non-significant) variation of the PRS-factor irrespective of QIRKS3 risk categories, albeit the CVD prevalence differs significantly between low- and high-risk categories (panel A). OR, odds ratio; CVD, cardiovascular disease; PRS, polygenic risk score.

## Figure S7


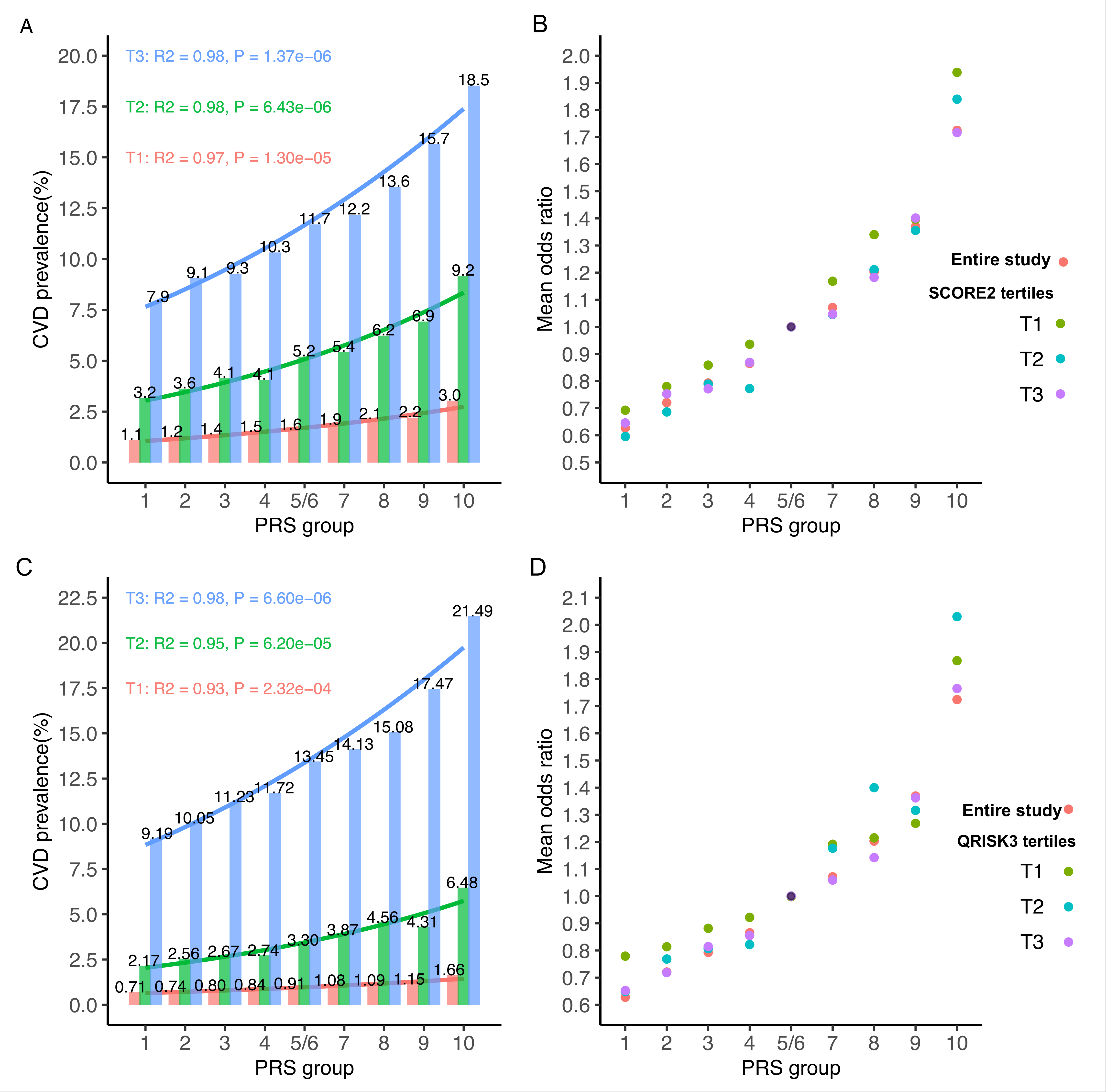


**Figure S7. CVD prevalence and PRS-factors in clinical risk tertiles of SCORE2 and QRISK3.** The left panels (A, C) show the prevalence of CVD along the PRS tenths in clinical risk tertiles (T1, T2, and T3) of SCORE2 and QRISK3. The distributions of CVD prevalence in risk tertiles fit into the Logit model with R^2^ > 0.9. Left panels (B, D) show CVD ORs in risk tertiles of SCORE2 and QRISK3 by PRS tenths of which fifth/sixth groups were taken as reference (OR = 1.0). The figure shows little (non-significant) variation of the CVD ORs conferred by the PRS irrespective of clinical risk tertiles. OR, odds ratio; CVD, cardiovascular disease; PRS, polygenic risk score.
